# Supplementary material for: Morphological changes in the tracheal system associated with light organs of the firefly Photinus pyralis (Coleoptera: Lampyridae) across life stages
Source: PLoS One. 2022 Jun 1;17(6):e0268112. doi: 10.1371/journal.pone.0268112 (PMC9159635; doi:10.1371/journal.pone.0268112)
Supplement: Attachment — Submitted filename: Response to Reviewers.docx [file pone.0268112.s001.docx]

Kristin N. Dunn

University of Florida

1881 Natural Area Drive

Gainesville, FL 32611

April 8, 2021

Dr. Feng Zhang

Academic Editor

*PLOS ONE*

Dear Dr. Zhang,

This letter is in response to the review of an original research article titled “Morphological changes in the tracheal system of the firefly *Photinus pyralis* (Coleoptera: Lampyridae) across life stages” submitted for publication consideration in *PLOS ONE*.

Addressing the points raised during the edit and review:

File Naming – File names have been checked.

Grant Information – The correct funding information is listed below.

Funding was provided by the National Science Foundation (https://www.nsf.gov/) M.A.B. DEB-1655936, a collaborative research grant with K.S.H. DEB-1655908 and S.M.B. DEB-1655981

Funding for the AMNH Micro-CT scanner was provided via an NSF instrumentation grant MRI-R2 0959384

H.W.H. was supported by a Richard Gilder Graduate School Fellowship

Data Availability – Raw micro-CT scan data have been deposited into Morphosource.org. Access links are below.

Project: https://www.morphosource.org/projects/000434376?locale=en

Larva tifs: https://www.morphosource.org/concern/media/000434523?locale=en

Pupa tifs: https://www.morphosource.org/concern/media/000434527?locale=en

Adult Male tifs: https://www.morphosource.org/concern/media/000434514?locale=en

Adult Female tifs: https://www.morphosource.org/concern/media/000434531?locale=en

Reference List – References remain the same as in the initial submission, with two exceptions: Citations 41-43 were missing from the in text citations. This has been corrected (Page 31). Two citations, 34 and 35, were reordered based on citation date within text citation (Page 40).

En dashes have been placed throughout the manuscript where appropriate.

Additional minor changes:

Bold type removed from title (Page 1).

Spelling correction (Page 5).

Duplicate table captions deleted (Pages 15, 23).

Spelling correction (Page 16).

General grammar throughout.

Although our reviewer suggested videos as supplementary data, we feel that our figures accurately portray the evidence we discuss in the article and that further imaging is not needed. Due to the time requirement of creating such videos, we feel it is in our best interest to move forward with the imaging as is but do appreciate this suggestion and will take this into consideration in future works.

Please address all correspondence concerning this manuscript to me at KristinDunn@ufl.edu

Thank you for your consideration.

Sincerely,

Kristin N. Dunn, MS

Doctoral Candidate, Department of Entomology and Nematology

University of Florida

(757) 619-7094

kristindunn@ufl.edu

Co-Authors:

Dr. Steven Davis

Division of Invertebrate Zoology

American Museum of Natural History

(785) 424-0810 sdavis@amnh.org

Hollister Herhold

Division of Invertebrate Zoology

American Museum of Natural History

hherhold@amnh.org

Dr. Kathrin Stanger-Hall

Department of Plant Biology

University of Georgia

(707) 542-1870

ksh@uga.edu

Dr. Seth M. Bybee

Department of Biology

Brigham Young University

(801) 422-3152

seth.bybee@byu.edu

Dr. Marc Branham

Department of Entomology and Nematology

University of Florida

(352) 273-3915 marcbran@ufl.edu
